# The mechanism and treatment of protein acylation modification in cardiovascular disease: a narrative review

**DOI:** 10.3389/fphar.2026.1866090

**Published:** 2026-06-30

**Authors:** Kaili Wang, Hanyi Wang, Jian Zhang, Jianing Liang, Qiaoyun Wen

**Affiliations:** 1 Department of Medical Management, Hunan Hospital of Integrated Traditional Chinese and Western Medicine, Changsha, China; 2 Department of Traditional Chinese Medicine, Hunan University of Chinese Medicine, Changsha, China; 3 Department of Basic Medicine, Heilongjiang University of Traditional Chinese Medicine, Harbin, China

**Keywords:** cardiovascular disease, inflammation, oxidative stress, post-translational modification, protein acylation, targeted therapy

## Abstract

Cardiovascular diseases are the leading causes of death and disability worldwide. Their initiation and progression involve multiple complex processes, including metabolic disorders, oxidative stress, inflammatory responses, and cell death. Protein acylation, a rapidly advancing field in post-translational modification research, dynamically regulates protein function, chromatin status, and metabolic signaling networks by covalently attaching various acyl groups to specific protein sites, thereby serving as a key molecular mechanism linking cellular metabolic states to cardiovascular pathology. This review systematically summarizes the mechanisms of protein acylation modifications, including acetylation, lactylation, 2-hydroxyisobutyrylation, and palmitoylation—in major cardiovascular diseases such as cardiac hypertrophy and heart failure, atherosclerosis, myocardial ischemia-reperfusion injury, and arrhythmias. It also provides an overview of recent therapeutic strategies targeting SIRT1, HDACs, acetyl-CoA, Snail1, NLRP3, and Khib-associated metabolic enzymes. Accumulating evidence indicates that maintaining histone acetylation homeostasis, activating SIRT1, inhibiting HDACs, or intervening in specific acylation sites can effectively alleviate myocardial injury, suppress inflammatory responses, and improve cardiac remodeling and repair, offering new insights for mechanistic research and precision therapy of cardiovascular diseases.

## 1 Introduction

Cardiovascular diseases are the leading cause of death and disability worldwide. Their occurrence and development involve multiple aspects such as metabolic disorders, oxidative stress, inflammatory responses, cell death, and tissue remodeling. According to survey research, approximately 127.9 million U.S. adults aged 20 and older (nearly 50%) had cardiovascular disease in 2020. In 2022, the disease caused over 941,000 deaths and remained the leading cause of death, with one myocardial infarction occurring every 40 s on average (Martin et al., 2025). Although the existing treatments have made significant progress in improving symptoms and reducing the risk of certain events, there are still insufficient and effective intervention methods for problems such as poor repair after myocardial infarction, continuous progression of heart failure, increased susceptibility to arrhythmias, and inflammatory myocardial damage.

As a rapidly advancing field in post-translational modification research in recent years, protein acylation is reshaping our understanding of the molecular mechanisms underlying cardiovascular diseases. Unlike the traditional view that primarily regarded acetylation as a nuclear histone modification, current studies have progressively demonstrated that protein acylation possesses broader biological significance. On the one hand, it participates in epigenetic regulation by modulating chromatin accessibility and transcriptional programs (Marmorstein and Zhou, 2022); on the other hand, it directly modifies metabolic enzymes, inflammasome components, ion channels, cytoskeletal proteins, and transcription factors, thereby influencing mitochondrial function, cellular energy supply, immune responses, and the stability of myocardial electrical activity (Sabari et al., 2017). More importantly, acylation modifications do not occur in isolation but are dynamically coupled with metabolic intermediates such as acetyl-CoA, lactate, and acyl-CoA, thus enabling the direct conversion of cellular metabolic states into changes in protein function and gene expression (Choudhary et al., 2014; Pietrocola et al., 2015). This feature indicates that protein acylation is not merely a concomitant phenomenon following disease onset, but may serve as a crucial mechanistic hub driving cardiovascular pathological processes.

Recent studies have shown that aberrant protein acylation is extensively involved in various cardiovascular diseases, including atherosclerosis, myocardial ischemia-reperfusion injury, myocardial infarction, cardiac hypertrophy, heart failure, and arrhythmias (Yan et al., 2022; Li et al., 2018). Imbalances in classical acetylation/deacetylation can affect histone modification status and non-histone protein functions, thereby regulating inflammatory responses, mitochondrial homeostasis, and cardiac remodeling. Meanwhile, novel modifications such as lactylation, palmitoylation, and 2-hydroxyisobutyrylation further reveal the critical roles of metabolic reprogramming, immune regulation, and inflammasome activation in cardiovascular pathology (Zhang et al., 2019; Linder and Deschenes, 2007; Huang et al., 2021a). Based on these findings, this review summarizes the main mechanisms of protein acylation in cardiovascular diseases and outlines related targets and therapeutic strategies, aiming to provide new insights for mechanistic research and precision therapy of cardiovascular diseases.

## 2 Overview of protein acylation modifications

### 2.1 Occurrence of protein acetylation modification

Protein acylation modification serves as a crucial regulatory link between cellular metabolic status and protein function, with its underlying mechanism reflecting the synergy between metabolic adaptation and molecular regulation. The core of protein acylation lies in the covalent transfer of metabolically derived activated acyl groups to specific amino acid residues. This dynamic process can occur via both enzymatic and non-enzymatic pathways (Ali et al., 2018; Simithy et al., 2017; Trefely et al., 2020; Kaczmarska et al., 2017). The enzyme-catalyzed route is a major mechanism of protein acylation. Lysine acylation is typically catalyzed by acyltransferases such as p300/CBP, GNAT, or MYST, whereas fatty acylation depends on DHHC family acyltransferases and N-myristoyltransferase. Meanwhile, the deacylation process is mediated by deacylases including the HDAC family and the Sirtuin family (Sabari et al., 2018). In addition, non-enzymatic acylation is also important in certain cellular compartments, particularly within the mitochondrial matrix, where highly reactive acyl-CoA molecules are abundant and the local environment is alkaline, leading to more pronounced non-enzymatic acylation of lysine residues (Jiang et al., 2018; Rardin et al., 2013). Modifications such as acetylation, succinylation, and malonylation in mitochondria are often closely associated with oxidative stress, metabolic disorders, and cellular dysfunction (Weinert et al., 2015). The mechanisms of protein acylation differ markedly across cellular compartments: acylation in the nucleus is mostly linked to chromatin structure and gene transcription regulation; mitochondria are enriched in lysine acylation, which is tightly coupled to metabolic enzyme activity; and lipid acylation primarily functions to enhance the hydrophobicity and membrane-anchoring properties of membrane proteins (Wagner and Payne, 2013). This subcellular heterogeneity allows acylation modifications to play broad roles in cell signaling, metabolic regulation, and pathological responses.

### 2.2 Types of protein acylation modifications

**FIGURE 1:**
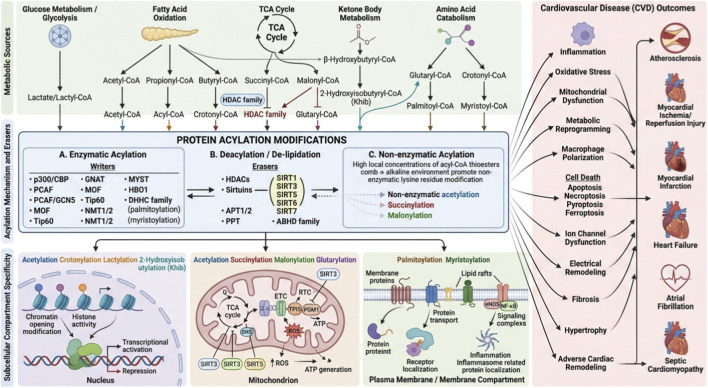
The mechanisms of different types of protein acylation modifications and their functional connections related to cardiovascular diseases. Protein acylation is dynamically regulated by cellular metabolism through the availability of multiple activated acyl donors, including acetyl-CoA, succinyl-CoA, malonyl-CoA, glutaryl-CoA, lactate-derived intermediates, and long-chain fatty acyl-CoA species. These metabolites drive both enzymatic and non-enzymatic acylation of proteins in distinct subcellular compartments. In the nucleus, acylation mainly regulates chromatin accessibility and transcriptional programs; in mitochondria, it modulates metabolic enzymes, redox balance, and energy production; and at membrane systems, lipid acylation controls membrane targeting, protein trafficking, and signaling complex assembly. Dysregulated protein acylation contributes to inflammation, oxidative stress, mitochondrial dysfunction, metabolic remodeling, ion channel instability, and multiple forms of cell death, thereby promoting atherosclerosis, myocardial ischemia/reperfusion injury, myocardial infarction, heart failure, atrial fibrillation, and septic cardiomyopathy.

Based on the receptor site and chemical properties, protein acylation can be broadly divided into two categories. One category is lysine acylation, which primarily targets lysine residues, including acetylation, propionylation, butyrylation, crotonylation, malonylation, succinylation, glutarylation, lactylation, β-hydroxybutyrylation, 2-hydroxyisobutyrylation, and benzoylation. The other category is lipid acylation, which modifies proteins with fatty acid chains, mainly including palmitoylation and myristoylation. The former is more involved in chromatin regulation, transcriptional control, and modulation of metabolic enzyme activity, whereas the latter primarily affects protein membrane anchoring, subcellular trafficking, and membrane-associated signal transduction (Allfrey et al., 1964; Choudhary et al., 2014), (Table 1; Figure 1).

**TABLE 1:** Main types, mechanisms and biological functions of protein acylation modifications.

Acylation type	Donor	Modification site	Writers/Erasers	Main biological functions	References
Acetylation (Kac)	Acetyl-CoA	Lysine ε-amino group; N-terminal α-amino group	Writers:p300/CBP, GNAT, MYST, NATs; Erasers: HDACs, SIRT1-3	Chromatin relaxation, transcriptional regulation, modulation of metabolic enzyme activity, inflammation and signal transduction	Ali et al. (2018), Zhao et al. (2010)
Propionylation (Kpr)	Propionyl-CoA	Lysine ε-amino group	Writers:p300/CBP, PCAF, GCN5; Erasers: SIRT1, SIRT3, SIRT5	Transcriptional activation, regulation of protein stability, control of energy metabolism	Ali et al. (2018), Zhao et al. (2010)
Butyrylation (Kbu)	Butyryl-CoA	Lysine ε-amino group	Writers:p300/CBP, PCAF, GCN5, HBO1; Erasers: SIRT1-3, SIRT6, SIRT7	Transcriptional activation, lipid metabolism, cell differentiation	Ali et al. (2018)
Crotonylation (Kcr)	Crotonyl-CoA	Lysine ε-amino group	Writers:p300/CBP, MOF; Erasers: SIRT1-3, HDAC1, HDAC3	Transcriptional activation, RNA processing, DNA damage repair, cell-cycle regulation	Ali et al. (2018)
Malonylation (Kmal)	Malonyl-CoA	Lysine ε-amino group	Writers: not fully defined; Erasers: SIRT5	Metabolic reprogramming, fatty acid metabolism, inflammation, and mitochondrial function regulation	Wagner and Payne (2013), Ali et al. (2018)
Succinylation (Ksucc)	Succinyl-CoA	Lysine ε-amino group	Writers: α-KGDH, KAT2A, HAT1, CPT1A; Erasers: SIRT5, SIRT7	Regulation of mitochondrial metabolism, transcriptional activation, maintenance of redox homeostasis	Wagner and Payne (2013), Ali et al. (2018)
Glutarylation (Kglu)	Glutaryl-CoA	Lysine ε-amino group	Writers: KAT2A, α-KADH; Erasers: SIRT5, SIRT7	Regulation of amino acid and mitochondrial metabolism, oxidative stress response, transcriptional regulation	Wagner and Payne (2013), Ali et al. (2018)
Lactylation (Kla)	Lactyl-CoA	Lysine ε-amino group	Writers: p300; Erasers: HDACs, Sirtuins	Links glycolysis/lactate metabolism with gene transcription, inflammatory responses, and cell phenotypic transition	Ali et al. (2018)
β-Hydroxybutyrylation (Kbhb)	β-Hydroxybutyryl-CoA; β-hydroxybutyrate	Lysine ε-amino group	Writers:p300/CBP; Erasers:HDAC1-3, SIRT3	Metabolic adaptation, ketone body signaling, transcriptional activation, redox homeostasis	Ali et al. (2018)
2-Hydroxyisobutyrylation (Khib)	2-Hydroxyisobutyryl-CoA	Lysine ε-amino group	Writers:p300/CBP, Tip60, Esa1p Erasers:HDAC1-3	Transcriptional activation, glycolysis/gluconeogenesis, amino acid biosynthesis	Wang J. et al. (2023), Xu et al. (2024)
Benzoylation (Kbz)	Benzoyl-CoA	Lysine ε-amino group	Writers: HBO1, p300/CBP, SAGA; Erasers: SIRT2, Hst2	Transcriptional regulation, glucose metabolism, ribosome biogenesis, and rRNA processing	Ali et al. (2018)
Palmitoylation	Palmitoyl-CoA	Cysteine residues (S-acylation)	Writers:DHHC1-23; Erasers: APT1/2, PPTs, ABHD family	Membrane anchoring, protein trafficking, assembly of receptor and inflammatory signaling complexes	Jiang et al. (2018); Resh (2016), Chamberlain and Shipston (2015)
Myristoylation	Myristoyl-CoA	N-terminal glycine residue	Writers: NMT1/2; Erasers: no well-defined classical erasers	Membrane targeting, signal transduction, recruitment of protein complexes	Jiang et al. (2018); Resh (2016)

#### 2.2.1 Lysine acylation

Lysine acylation is the most extensively studied class of protein acylation. Its common feature is the attachment of an acyl group to the ε-amino group of the lysine side chain, thereby neutralizing the original positive charge or, in the case of certain dicarboxyl acylations, causing charge reversal, which in turn affects protein conformation, stability, and function. Most lysine acylations utilize the corresponding acyl-CoA or activated metabolic intermediates as donors and are catalyzed by broad-spectrum or specific acyltransferases such as p300/CBP, GNAT, and MYST, while deacylation primarily depends on the HDAC and Sirtuin families (Zhao et al., 2010). In addition to enzymatic pathways, in cellular compartments rich in reactive acyl-CoAs and with a locally alkaline environment, such as mitochondria, certain lysine acylations can also occur non-enzymatically; therefore, their modification levels often exhibit both enzyme activity dependence and metabolic state sensitivity (Wagner and Payne, 2013; Weinert et al., 2013). Among these, acetylation is the earliest studied and most classical form of lysine acylation. Its donor is acetyl-CoA, and it is primarily catalyzed by lysine acetyltransferases, with deacetylation mediated by HDACs and Sirtuins. Acetylation weakens the interaction between lysine and DNA or other negatively charged molecules, thus playing a central role in histone modification, chromatin accessibility, transcriptional activation, and the regulation of non-histone protein functions (Kouzarides, 2007). Propionylation, butyrylation, and crotonylation, which are structurally similar to acetylation, use propionyl-CoA, butyryl-CoA, and crotonyl-CoA as donors, respectively, and are often catalyzed by acyltransferases with substrate promiscuity (Chen et al., 2007; Liu et al., 2009; Tan et al., 2011). These modifications, owing to differences in acyl chain length, hydrophobicity, and spatial rigidity, do not exert identical effects on protein local conformation and molecular interactions as acetylation. Among them, crotonylation, characterized by a conjugated double bond and a planar structure, has garnered particular attention in transcriptionally active regions and is recognized as an important epigenetic activation mark beyond acetylation (Peng et al., 2011). In contrast to the above neutral short-chain acylations, malonylation, succinylation, and glutarylation belong to dicarboxyl acylations, with malonyl-CoA, succinyl-CoA, and glutaryl-CoA serving as their respective acyl donors (Zhang et al., 2011; Tan et al., 2014). These modifications not only increase the volume of the lysine side chain but also significantly alter its charge properties, thereby often exerting stronger effects on metabolic enzyme activity, protein complex assembly, and mitochondrial function. Because the corresponding acyl-CoAs are widely present in fatty acid metabolism, the tricarboxylic acid cycle, and amino acid catabolic pathways, these modifications are particularly common in metabolically active mitochondria. SIRT5 is currently the best-characterized demalonylase, desuccinylase, and deglutarylase, playing a critical role in maintaining the functional homeostasis of metabolic proteins.

In recent years, novel metabolism-related acylations have been continuously identified, further expanding the biological implications of protein acylation. Lactylation directly links enhanced glycolysis and lactate accumulation to epigenetic regulation, occurs on both histones and non-histone proteins, and is particularly prominent under conditions of hypoxia, inflammation, and immune cell activation (Zhang et al., 2019). β-Hydroxybutyrylation reflects an active ketone body metabolic state, often increases during fasting, sustained exercise, and diabetes, and is considered an important epigenetic marker of energy stress adaptation (Xie et al., 2016). 2-Hydroxyisobutyrylation is a novel type of lysine acylation with a bulky, branched structure, widely distributed among metabolic and translation-related proteins, and may exert more pronounced effects on protein conformation and enzyme active centers (Dai et al., 2014). In cardiovascular research, studies have shown that Khib modification in atrial tissue can affect the function of the glycolytic enzyme HXK1 and further regulate energy metabolism and KATP channel function, suggesting its potential involvement in the development and progression of atrial fibrillation (Hou et al., 2025). Benzoylation, an aromatic acylation form, contains an aromatic ring structure that theoretically imparts unique spatial and hydrophobic properties to proteins. Currently, its substrate spectrum, physiological sources, and pathological significance remain to be further elucidated (Huang et al., 2021b). Collectively, these novel lysine acylations demonstrate that protein acylation is not only part of classical epigenetic modifications but also a key mediator through which cellular metabolic status directly participates in the regulation of protein function.

#### 2.2.2 Lipid acylation

Unlike lysine acylation, which primarily targets the lysine side chain, lipid acylation involves the covalent attachment of long-chain fatty acids to the N-terminus or cysteine residues of proteins, thereby enhancing protein hydrophobicity and membrane affinity. This type of modification plays an important role in membrane protein localization, vesicular trafficking, receptor signal transduction, and intercellular communication, and is particularly relevant to ion channel function, receptor activation, and inflammatory signaling in the cardiovascular system. Palmitoylation generally refers to S-palmitoylation, i.e., the attachment of palmitic acid via a thioester bond to a cysteine residue. Its donor is palmitoyl-CoA, and the primary writers are DHHC family palmitoyltransferases (Linder and Deschenes, 2007). Unlike most lysine acylations, S-palmitoylation is highly reversible, as it can be removed by members of the APT, PPT, and ABHD families, thus representing an important mechanism for dynamically regulating protein membrane localization and signaling complex assembly. Many receptors, ion channels, G proteins, and inflammation-related proteins are regulated by palmitoylation, suggesting its significance in membrane signal transduction, inflammatory amplification, and maintenance of electrophysiological homeostasis. Furthermore, in cardiovascular disease-related studies, Cys202 of cyclophilin D can undergo various post-translational modifications and is involved in cardioprotection, indicating that cysteine site modifications play an important regulatory role in mitochondrial function and myocardial injury responses (Amanakis et al., 2021). Myristoylation primarily refers to N-myristoylation, in which N-myristoyltransferase uses myristoyl-CoA as a donor to attach myristic acid to the N-terminal glycine residue of a protein. This modification typically occurs early in post-translational processing, forming a stable amide bond, and is generally considered relatively stable or even nearly irreversible. Although myristoylation itself confers only limited membrane affinity to the protein, it often acts in concert with polybasic regions or palmitoylation to jointly determine the precise membrane localization and functional status of the protein. Therefore, myristoylation can be regarded as an important preparatory step for membrane signal transduction and protein subcellular distribution.

## 3 The mechanism of protein acylation modification in cardiovascular diseases

### 3.1 Myocardial hypertrophy and heart failure

Acetylation was recognized as one of the most thoroughly studied acylation modifications in cardiac hypertrophy and heart failure (HF), and both histone acetylation-mediated transcriptional reprogramming and direct regulation of metabolic, contractile, and stress signals by non-histone acetylation were involved (McKinsey and Olson, 2004; Hohl et al., 2013; Morales et al., 2016). Early in pressure overload, histone acetylation imbalance was observed, and active markers such as H3K9ac, H3K14ac, and H3K27ac were found to promote the transcription of hypertrophy-related genes and the reactivation of the fetal gene program, thereby driving cardiac remodeling (Papait et al., 2013; Gallo et al., 2008). Consistently, inhibition of histone acetyltransferase activity was shown to attenuate cardiac hypertrophy, suggesting that hyperacetylation was an important epigenetic basis for pathological remodeling (R et al., 2010). In contrast, deacetylases were demonstrated to exhibit stronger isoform- and stage-specificity in this process. HDAC5 was significantly upregulated under pressure overload and in decompensated heart failure, and was associated with fibrosis and deterioration of cardiac function. In a transverse aortic constriction (TAC) mouse model and in H9C2 cardiomyocytes treated with angiotensin II (Ang II), inhibition of HDAC5 was found to activate the ERK/EGR1 signaling pathway, thereby upregulating the expression of myocyte enhancer factor 2A (MEF2A), which consequently improved cardiac function, attenuated ventricular remodeling, and reduced cardiomyocyte hypertrophy (Zhu et al., 2023; Bai et al., 2021; Backs et al., 2006). In addition to nuclear transcriptional regulation, aberrant acetylation of mitochondrial proteins was identified as another key metabolic feature of heart failure (HF). In conditions such as obesity, diabetes, and heart failure with preserved ejection fraction (HFpEF), cardiac mitochondria were found to be hyperacetylated, suggesting a close relationship between acetylation imbalance and metabolic reprogramming (Fillmore et al., 2014; Horton et al., 2019). SIRT3, as the primary mitochondrial deacetylase, was recognized as a key node in maintaining cardiac energy homeostasis. In Myh6-Cre transgenic mouse hearts with SIRT3 knockout and in H9c2 myogenic cells, SIRT3 deficiency was shown to increase p53 acetylation, leading to downregulation of GPX-4 and elevation of 4-HNE, thereby inducing ferroptosis and promoting myocardial fibrosis. Conversely, SIRT3 overexpression or inhibition of p53 acetylation was demonstrated to alleviate the aforementioned ferroptosis and fibrosis (Su et al., 2023; Gao et al., 2023). Although the effects of acetylation at different sites on fatty acid oxidation enzyme activities were observed to be context-dependent, it was widely accepted that mitochondrial acetylation imbalance promotes abnormal energy utilization and accelerates HF progression. Aberrant acetylation was also found to further affect cardiomyocyte function and interstitial remodeling. In a left anterior descending (LAD) ligation-induced ischemic HF rat model and in OGD/R-treated H9c2 cells, propanol hormone (CAL) was found to upregulate SIRT1 and, in a SIRT1-dependent manner, promote FOXD3 deacetylation and nuclear translocation, thereby upregulating SERCA2a, which consequently increased ejection fraction and inhibited myocardial fibrosis. Moreover, in failing hearts from humans, mice, and pigs, reduced SIRT1 levels were shown to result in p300-mediated hyperacetylation of SERCA2a at lysine 492 (K492), and this acetylation inhibited SERCA2a activity by interfering with ATP binding. Activation of SIRT1 was demonstrated to promote K492 deacetylation and restore SERCA2a function, thereby ameliorating heart failure (Chen et al., 2024; Gorski et al., 2019). In contrast, increased acetylation of Drp1 was reported to promote excessive mitochondrial fission and cardiomyocyte death, driving the deterioration of cardiac function (Song et al., 2017). In a rat model of cardiac hypertrophy induced by transverse aortic constriction (TAC) and in AC16 and HCM cardiomyocytes treated with angiotensin II (Ang II), high expression of SIRT1 was found to inhibit Drp1 acetylation, thereby reducing NLRP3 inflammasome activation and upregulating the antioxidant factors HO-1 and Nrf2, which consequently alleviated cardiac hypertrophy and fibrosis in a dose-dependent manner (Ren et al., 2021; Wang et al., 2025).

In addition to acetylation, the role of mitochondrial lysine acylation modifications in heart failure (HF) was gradually recognized, among which succinylation was considered the most representative. Succinylation was found to significantly alter the charge status of lysine residues, thereby exerting a strong impact on protein conformation, complex stability, and enzymatic activity (Rardin et al., 2013; Park et al., 2013). SIRT5 was identified as the primary mitochondrial desuccinylase, also possessing demalonylase and deglutarylase activities, and was recognized as a key regulator of mitochondrial acylation homeostasis (Xiao et al., 2021; Wu et al., 2024). It was demonstrated that in mice with myocardial fibrosis and heart failure induced by transverse aortic constriction (TAC) as well as in HL-1 cells treated with high glucose, upregulation of SIRT5 expression promoted the desuccinylation of IDH2, thereby improving mitochondrial energy metabolism, maintaining mitochondrial homeostasis, and inhibiting oxidative stress and inflammatory responses, which consequently alleviated myocardial fibrosis and improved cardiac function (Chang et al., 2021). Deficiency of SIRT5 was found to increase the succinylation of the mitochondrial trifunctional enzyme subunit alpha (ECHA), which led to the inhibition of its enzymatic activity, impairment of fatty acid β-oxidation, and reduction of ATP production efficiency, thereby inducing insufficient energy supply and ventricular remodeling. Because adult myocardium was known to primarily rely on fatty acid oxidation for energy production, the disturbance of mitochondrial metabolism caused by succinylation imbalance was considered to promote the transition of hypertrophic myocardium from a compensated state to decompensated HF (Doenst et al., 2013; Fukushima and Lopaschuk, 2016). Notably, malonylation and glutarylation were also shown to be mainly enriched in mitochondrial metabolic proteins; although direct evidence for their involvement in HF remained relatively limited, given the central role of SIRT5 in fatty acid oxidation, oxidative phosphorylation, and redox homeostasis, these dicarboxyl acyl modifications were considered to potentially participate in the loss of metabolic flexibility and in mitochondrial dysfunction of the heart (Belosludtsev et al., 2020). Therefore, from the perspective of metabolic reprogramming, multiple SIRT5-centered mitochondrial deacylation reactions were suggested to collectively influence the energy compensatory capacity of hypertrophic myocardium and the progression of heart failure.

2-Hydroxyisobutyrylation (Khib) has recently been recognized as a lysine acylation modification that is closely associated with cellular metabolic status, particularly glucose metabolism (Zhang N. et al., 2021; Huang et al., 2022). Although the investigation of Khib in the cardiovascular system was still limited, it was demonstrated that in a mouse model of cardiac hypertrophy induced by transverse aortic constriction (TAC), the acyltransferase p300 was found to cause a reduction in pyruvate dehydrogenase (PDH) activity by inducing Khib modification on the pyruvate dehydrogenase complex subunit DLD, thereby aggravating cardiac hypertrophy in the TAC mouse model (Ni et al., 2022). Pathological cardiac hypertrophy was characterized by typical metabolic reprogramming, manifested as enhanced glycolysis and reduced mitochondrial oxidative capacity, leading to decreased glucose utilization efficiency. For instance, in HCT116 and HEK293T cells, p300 was shown to exert its lysine 2-hydroxyisobutyryltransferase activity to introduce Khib modifications on glycolytic enzymes, specifically at the K281 site of ENO1 and on PFKM, thereby enhancing the activities of these enzymes, promoting glycolysis, and consequently causing glucose depletion-induced cell death (Huang et al., 2018; Murashige et al., 2020). In the TAC-induced cardiac hypertrophy model, elevated levels of pyruvate and lactate in myocardial tissue, along with a decreased content of acetyl-CoA and reduced PDH activity, were observed, suggesting that the conversion of glycolytic end products to mitochondrial oxidative metabolism was impaired. During this process, dysfunction of the PDH complex was recognized as a critical link between enhanced glycolysis and impaired oxidative metabolism (Zhao et al., 2024). Previous studies have shown that dihydrolipoamide dehydrogenase (DLD), as an essential component of the PDH complex, could be regulated by its modification status, which significantly influenced the efficiency of pyruvate oxidation. Under pressure overload conditions, aberrant Khib modification was found to inhibit PDH-mediated pyruvate oxidation, possibly by affecting the conformation and activity of key metabolic enzymes such as DLD and DLAT, thereby promoting lactate accumulation and inefficient glucose metabolism (Wang J. et al., 2023). This metabolic disturbance was considered to not only limit ATP production but also exacerbate acidosis, oxidative stress, and hypertrophic signaling activation, ultimately driving pathological cardiac remodeling.

**FIGURE 2:**
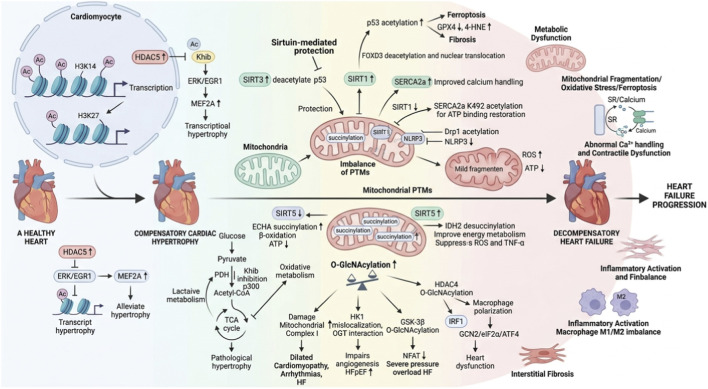
Role of protein acylation-related modifications in cardiac hypertrophy and heart failure. Under pressure overload, metabolic abnormalities, and inflammatory stimulation, histone H3 acetylation (at H3K9ac, H3K14ac, and H3K27ac) was activated, which facilitated chromatin opening and enhanced the expression of hypertrophy-related genes. Concurrently, upregulation of HDAC5 was associated with fibrosis and functional deterioration, and its inhibition was shown to improve ventricular remodeling via the ERK/EGR1 signaling pathway. Within mitochondria, acetylation imbalance and reduced function of the SIRT3/SIRT1 axis were found to lead to metabolic disturbances, ferroptosis, and inflammatory activation, in which SIRT1 was demonstrated to regulate SERCA2a deacetylation and improve calcium handling, whereas SIRT3 deficiency was shown to trigger ferroptosis and fibrosis. Moreover, SIRT5 was found to promote mitochondrial metabolic homeostasis through desuccinylation, and its deficiency was shown to exacerbate fatty acid β-oxidation impairment and ATP reduction. Metabolic reprogramming, mediated by p300-catalyzed 2-hydroxyisobutyrylation, was demonstrated to inhibit pyruvate oxidation by suppressing PDH complex activity, leading to lactate accumulation and aggravated hypertrophy. Meanwhile, O-GlcNAcylation was shown to exhibit bidirectional regulatory effects: excessive O-GlcNAcylation was found to exacerbate pathology, whereas moderate levels were protective, and its influence on inflammation and fibrosis was mediated through macrophage polarization.

Although O-GlcNAcylation was not classified as a classical lysine acylation, it was recognized as a metabolism-sensitive post-translational modification that exhibited extensive crosstalk with acylation networks such as acetylation, and was also considered of great importance in heart failure (HF) (Hu et al., 2025; Ding et al., 2025). Available evidence suggested that the role of O-GlcNAcylation in HF displayed clear biphasic characteristics. In a mouse model of heart failure with preserved ejection fraction (HFpEF) and in mouse endothelial cells lacking the mitochondrial binding domain of hexokinase 1 (HK1), mislocalization of HK1 to mitochondria was found to enhance its interaction with O-linked N-acetylglucosamine transferase (OGT), thereby increasing protein O-GlcNAcylation levels, which consequently led to impaired angiogenesis and promoted the development of HFpEF. Furthermore, in transgenic mouse models with cardiac-specific overexpression of OGT or OGA, elevated OGT was shown to cause an increase in O-GlcNAcylation, which subsequently impaired mitochondrial complex I activity, leading to severe dilated cardiomyopathy, ventricular arrhythmias, and premature death. In contrast, OGA overexpression was demonstrated to reduce O-GlcNAcylation and restore complex I activity, thereby exerting a protective effect against pressure overload-induced pathological cardiac remodeling and heart failure (Tatekoshi et al., 2025; Umapathi et al., 2021). It was shown that O-GlcNAcylation of GSK-3β led to inactivation of NFAT, thereby aggravating pressure overload-induced heart failure (HF), suggesting that this modification accelerated the decompensation process by altering stress adaptation signaling (Facundo et al., 2020). However, O-GlcNAcylation was not always pathogenic, as its effects were found to be significantly substrate-dependent. O-GlcNAcylation of HDAC4 was demonstrated to exert a protective effect on the diabetic heart and to alleviate the development of HF, indicating that O-GlcNAc modification of specific proteins might participate in compensatory protective responses (Ramirez-Correa et al., 2021). Of greater interest, it was recently demonstrated that OGT-mediated O-GlcNAcylation regulated macrophage polarization by acting on IRF1. In mice with TAC-induced HF, inhibition of OGT was shown to promote the conversion of macrophages from a pro-inflammatory phenotype to an anti-inflammatory phenotype, thereby alleviating inflammatory responses, inhibiting fibrosis, and improving cardiac function (Zhang Y. et al., 2024). Similarly, in neonatal rat ventricular myocytes (NRVMs) and in cardiomyocyte-specific inducible OGT knockout mice, inhibition or deletion of OGT was found to lead to reduced O-GlcNAcylation levels, which subsequently activated the GCN2/eIF2α/Atf4 signaling pathway, resulting in suppression of protein synthesis, myocardial dysfunction, and heart failure. Notably, inhibition of this signaling pathway by ISRIB was shown to significantly delay the heart failure induced by OGT deficiency. These findings indicated that O-GlcNAc-related modifications not only acted on the intrinsic homeostasis of cardiomyocytes but also influenced the progression of HF by remodeling the immune microenvironment (Figure 2).

### 3.2 Atherosclerosis and vascular endothelial function

In atherosclerosis, an imbalance in acetylation/deacetylation homeostasis was identified as one of the most well-defined acylation abnormalities, and was found to be involved in multiple key processes, including lipid deposition, inflammatory activation, endothelial dysfunction, and smooth muscle remodeling (Lv et al., 2022; Min et al., 2024). Among them, HDAC-mediated deacetylation abnormalities were considered to be particularly important, and distinct HDAC isoforms were shown to exhibit clear cell-type specificity and stage dependence. Previous studies demonstrated that hyperhomocysteinemia promoted macrophage lipid accumulation and foam cell formation through upregulation of HDAC1, reduction of H3K9ac, and inhibition of miR-34a; simultaneously, HDAC3 was also shown to suppress cholesterol efflux via the lncRNA KCNQ1OT1/miR-452–3p/ABCA1 pathway, thereby further exacerbating lipid deposition (Li et al., 2021; Liu et al., 2023). At the inflammatory level, increased myeloid HDAC2 activity was closely associated with high-calorie diet-related monocyte dysfunction and vascular inflammation. Moreover, inhibition of the activation of HDAC2 by Roundabout guidance receptor 4 (Robo4) was found to reduce the release of the inflammatory cytokines IL-1β and TNF-α through restoration of redox homeostasis (Kashio et al., 2021). Therefore, HDAC abnormalities were shown to, on the one hand, promote foam cell formation, and on the other hand, amplify chronic inflammatory responses, jointly driving plaque progression.

In addition to epigenetic regulation at the histone level, non-histone acetylation was also found to be directly involved in the maintenance of vascular endothelial homeostasis. The acetylation status of eNOS was shown to influence its binding to calmodulin and its capacity to generate NO. Specifically, HDAC3-mediated deacetylation was demonstrated to inhibit eNOS activity and reduce endothelium-dependent relaxation, whereas promoting eNOS acetylation was found to help preserve the vasoprotective effect (Zhao et al., 2020; Zhang Q. et al., 2021). Furthermore, SIRT6 was shown to inhibit the transendothelial transport of LDL by regulating Caveolin-1 deacetylation and autophagy, suggesting that acetylation imbalance could also participate in early atherogenesis by affecting lipoprotein transport (Yang et al., 2022). On the other hand, the acetyltransferase KAT7 was reported to play an important role in maintaining the stability of the endothelial phenotype and the transcription of VEGFR-2, indicating that acetylation not only regulated vasodilation but also contributed to vascular repair and endothelial integrity (Xu et al., 2021). Acetylation abnormalities were also deeply involved in the phenotypic switching of vascular smooth muscle cells (VSMCs) and intimal remodeling. Multiple HDAC isoforms were found to be upregulated upon mitogen stimulation and vascular injury. In an atherosclerosis mouse model (ApoE-deficient mice fed a high-fat diet) and in TNF-α-treated human umbilical vein endothelial cells (HUVECs), HDAC11 was shown to promote cell pyroptosis and inflammation through the NLRP3/caspase-1/GSDMD and caspase-3/GSDME pathways by reducing the acetylation level of the transcription factor ERG, thereby exacerbating atherosclerotic lesions. Conversely, knockdown of HDAC11 or GSDME was demonstrated to significantly alleviate pyroptosis (Yao et al., 2022; Gao et al., 2020). Another study showed that HDAC inhibition induced acetylation of C/EBPα, which subsequently upregulated PPARγ and its downstream cholesterol efflux transporters ABCA1/ABCG1, thereby reducing foam cell formation and suppressing TNFα and IL-1β, leading to attenuation of atherosclerotic lesions in ApoE mice (Yao et al., 2022, Gao et al., 2020). In addition to histone modifications, the acetyltransferase MYST1 was reported to promote the switch of VSMCs from a contractile to a synthetic phenotype by enhancing the acetylation-mediated stability of KLF4, thereby accelerating injury-induced intimal hyperplasia (Zhou et al., 2024). These findings indicated that acetylation could both regulate proliferative programs by remodeling chromatin status and directly drive vascular structural remodeling by affecting the stability of key transcription factors.

**FIGURE 3:**
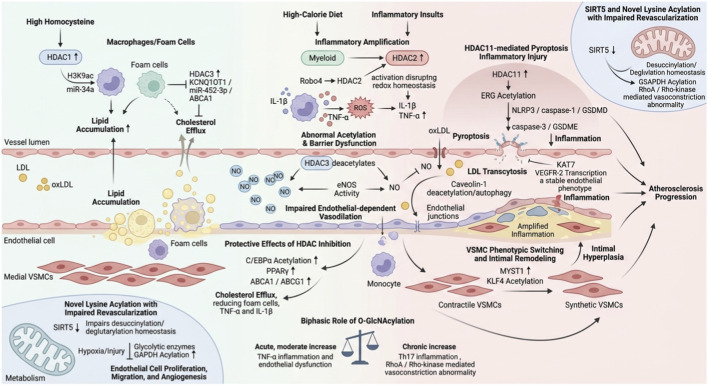
Schematic overview of acylation-related mechanisms in atherosclerosis and vascular endothelial dysfunction. Activation of acetylation within cells and regions was found to result in increased levels of H3K9ac, H3K14ac, and H3K27ac, as well as upregulation of HDAC5, which together led to cardiomyocyte hypertrophy and fibrosis. The energy metabolic disorder and reduced SIRT expression caused by the hyperacetylated state within mitochondria were shown to promote ferroptosis, reactive oxygen species (ROS) production, and NLRP3 inflammasome activation. Succinylation dysregulation was demonstrated to lead to impaired fatty acid β-oxidation and decreased ATP production. Khib-mediated inhibition of PDH was found to affect glucose metabolic reprogramming, as was the complex biphasic modulation of O-GlcNAcylation in regulating mitochondrial function, NFAT inactivation, macrophage polarization, and fibrosis.

In addition to classical acetylation, the metabolism-sensitive modification O-GlcNAcylation and various novel lysine acylations were also found to provide new mechanistic dimensions for the study of atherosclerosis. O-GlcNAcylation was shown to exhibit biphasic effects in the vascular system. An acute increase was found to alleviate TNF-α-induced inflammatory vascular hyporeactivity and endothelial dysfunction, whereas a chronic elevation was demonstrated to exacerbate vascular dysfunction by promoting the Th17 inflammatory program and enhancing RhoA/Rho-kinase-mediated vasoconstriction (Lima et al., 2021; Swamy et al., 2020). Existing studies suggested that SIRT5-dependent desuccinylation/deglutarylation homeostasis was closely associated with mitochondrial metabolism, redox balance, and inflammatory regulation. In hypoxia-treated HUVECs and in mouse models, downregulation of SIRT5 expression was shown to reduce glycolytic enzyme levels and increase the acrylation modification of GAPDH, thereby inhibiting endothelial cell proliferation, migration, and angiogenesis, which consequently exacerbated ischemic injury. Modulation of SIRT5 activity was suggested to potentially promote angiogenesis (Chen et al., 2018; Wei et al., 2023; Chen et al., 2026). Although direct evidence for these novel modifications in atherosclerosis remained limited, they were considered to potentially serve as important complementary mechanisms for understanding the development and progression of vascular diseases (Figure 3).

### 3.3 Myocardial ischemia-reperfusion injury

In myocardial ischemia/reperfusion injury (MIRI), acylation-related post-translational modifications were recognized as an important regulatory layer linking metabolic disturbances, oxidative stress, mitochondrial damage, and programmed cell death. Among these, an imbalance in lysine acetylation/deacetylation was considered one of the best-established mechanisms (Davidson et al., 2019; Hausenloy and Yellon, 2020). Acetylation levels were determined jointly by acetyltransferases and deacetylases, and their changes were found to not only affect histone modifications and stress-related transcriptional programs but also directly regulate the stability, enzymatic activity, and protein-protein interactions of various non-histone substrates, thereby determining the extent of cardiomyocyte injury after reperfusion. In cardiomyocytes subjected to oxygen-glucose deprivation/reoxygenation and in rats with left anterior descending coronary artery ligation-induced MIRI, lysine acetyltransferase 5 (KAT5) was shown to promote STUB1 transcription through acetylation modification, which in turn facilitated the ubiquitination and degradation of LATS2, activated the YAP/β-catenin pathway, and consequently inhibited NLRP3-mediated pyroptosis of cardiomyocytes (Liu C. et al., 2024). Overall, the roles of the HDAC family in MIRI were found to be markedly heterogeneous. Activation of certain classical HDACs was associated with exacerbated reperfusion injury, whereas NAD^+^-dependent deacetylases such as SIRT1 and SIRT3 predominantly exerted protective effects (Bugyei-Twum et al., 2021). It was demonstrated that HDAC inhibition alleviated oxidative stress and myocardial injury during the reperfusion phase by enhancing the expression of the antioxidant genes FoxO3a and its downstream targets SOD2 and catalase in rats subjected to MIRI, suggesting that histone acetylation remodeling was involved in the activation of the antioxidant transcriptional network (Xie et al., 2020). Meanwhile, in MIRI, SIRT1 expression was found to be significantly decreased, acetylation levels were elevated, and the activation of the AMPK/PGC-1α signaling pathway was inhibited. Consequently, the expression of NRF1, TFAM, and FOXO1 was downregulated, leading to extensive myocardial infarction. In contrast, activation of SIRT1 was shown to improve mitochondrial energy metabolism, increase the levels of NAD^+^, ATP, and SOD, and reduce the levels of ROS, MDA, and the AMP/ATP ratio, indicating that SIRT1-mediated deacetylation was an important basis for endogenous anti-ischemic protection (Tian et al., 2019).

At the mitochondrial level, acetylation homeostasis plays a particularly critical role in MIRI. Mitochondria are central hubs for Ca^2+^ overload, ROS burst, and abnormal opening of the mitochondrial permeability transition pore (mPTP) during reperfusion, and SIRT3, as the major mitochondrial deacetylase, occupies a central position in maintaining this homeostasis (Porter et al., 2021; Ruiz-Meana et al., 2020). SIRT3 deficiency exacerbates cardiac dysfunction and increases infarct size after I/R, and enhances mitochondrial sensitivity to Ca^2+^-induced mPTP opening. Among the key targets, cyclophilin D (CyPD) is one of the most directly evidenced proteins. In a hypoxia/reoxygenation (H/R)-induced injury model of H9c2 cardiomyocytes, it was shown that upregulation of SIRT3 expression promoted the lysine deacetylation of CypD, thereby protecting mitochondrial function, maintaining the activities of Na^+^/K^+^-ATPase and Ca^2+^-ATPase, reducing lactate dehydrogenase (LDH) release, and restoring nitric oxide (NO) levels, which consequently alleviated cell injury. (Wu et al., 2023). Furthermore, the acylation status of CyPD is not limited to acetylation; S-acylation/deacylation at the conserved C202 site, dynamically coupled with oxidative modifications, also influences CyPD conformation and interaction networks, thereby collectively determining the mPTP opening threshold (Amanakis and Murphy, 2022). This indicates that multi-level acylation regulation centered on CyPD may constitute an important molecular switch for mitochondrial injury in MIRI. In addition to direct mitochondrial damage, acetylation is also closely coupled with myocardial metabolic reprogramming and epigenetic responses. Ischemia-reperfusion leads to a decrease in acetyl-CoA levels, thereby limiting the supply of histone acetylation donors, reducing active markers such as H3K9ac, H3K14ac, and H3K27ac, and inhibiting the transcriptional activation of antioxidant genes (Lei et al., 2022). Studies have shown that medium-chain fatty acid metabolic substrates can restore Kat2a-dependent histone acetylation by increasing acetyl-CoA generation, elevate H3K9ac levels at the promoter regions of antioxidant genes such as HO1, NQO1, and SOD2, thereby reducing ROS accumulation and cell injury (Zhang et al., 2023). This finding suggests that histone acetylation is not an isolated chromatin event but is deeply embedded in the myocardial energy metabolism network during reperfusion, with acetyl-CoA serving both as an indicator of metabolic status and as a key epigenetic substrate determining whether antioxidant transcriptional programs can be effectively initiated.

In recent years, the role of non-histone acetylation in MIRI has also received increasing attention. It was shown that in rats subjected to left anterior descending coronary artery ligation and reperfusion (MIRI) as well as in H9c2 cells treated with hypoxia/reoxygenation (H/R), downregulation of p300/CBP-associated factor (PCAF) alleviated cardiomyocyte apoptosis, inflammation, and oxidative stress, and improved MIRI, which was achieved through the inhibition of NF-κB signaling pathway activation (Qiu et al., 2021). This mechanism indicates that aberrant acetylation not only affects classical transcription factors, metabolic enzymes, and mitochondrial pore proteins but may also reshape the myocardial response to ischemic stress through a novel axis of “acetylation–alternative splicing–mitochondrial adaptation.” Furthermore, studies have shown that sevoflurane postconditioning enhances OGT-mediated O-GlcNAcylation, inhibits RIPK3/MLKL-dependent necroptosis, and thereby attenuates reperfusion injury; conversely, inhibition of OGT reverses these protective effects, indicating that O-GlcNAc modification participates in cardioprotection by regulating the assembly of death signaling complexes (Wang X. et al., 2023).

**FIGURE 4:**
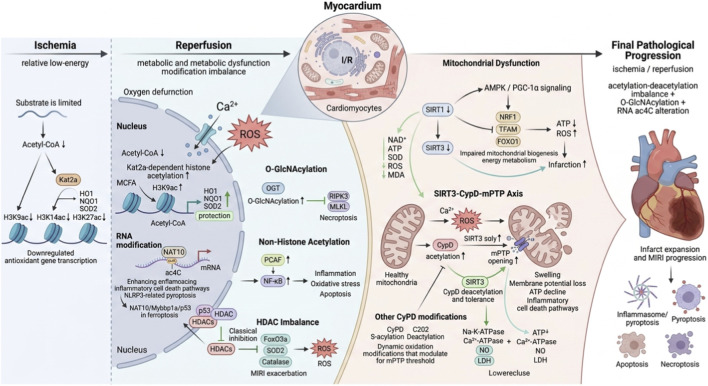
Acylation-related remodeling in MIRI. In the nucleus, ischemia/reperfusion was shown to inhibit acetyl-CoA production, leading to a decrease in histone acetylation levels and suppression of the transcriptional program of antioxidant genes (HO1, NQO1, SOD2). Partial restoration of Kat2a-dependent histone acetylation and the antioxidant transcriptional network was achieved by increasing acetyl-CoA production. KAT5 was demonstrated to activate the YAP/β-catenin pathway and inhibit NLRP3 inflammasome activity by enhancing STUB1-mediated degradation of LATS2, thereby forming a protective acetylation axis. Members of the HDAC family were found to exhibit heterogeneity: activation of classical HDACs was shown to promote injury, whereas SIRT1 was demonstrated to maintain mitochondrial homeostasis and suppress injury through the antioxidant transcription factor FoxO3a and its downstream AMPK/PGC-1α signaling pathway. Within mitochondria, SIRT3 was shown to regulate CypD via deacetylation, inhibit mPTP opening, and thereby ameliorate mitochondrial dysfunction triggered by calcium overload and ROS burst. In the cytoplasmic signaling domain, the PCAF-NF-κB pathway was identified as a promoter of inflammation and apoptosis, while OGT-mediated O-GlcNAcylation was demonstrated to provide protective regulation against necroptosis. Furthermore, NAT10-mediated RNA ac4C modification was found to be associated with NLRP3-mediated pyroptosis, further revealing the role of the acylation network in RNA-level regulation.

In addition to protein-level modifications, RNA acetylation provides a new layer for MIRI research. In recent years, NAT10-mediated N4-acetylcytidine (ac4C) modification has been implicated in inflammatory cell death and tissue damage following myocardial I/R (Zhang H. et al., 2024; Liu Y. et al., 2024). Related studies suggest that inhibition of NAT10-dependent ac4C modification alleviates NLRP3-associated pyroptosis and ameliorates myocardial injury in both H/R and *in vivo* I/R models; moreover, the NAT10/Mybbp1a/p53 axis may also be involved in the regulation of ferroptosis. Unlike protein acetylation, which primarily affects enzyme activity and protein-protein interactions, RNA acetylation focuses more on the post-transcriptional fine-tuning of mRNA stability and translational fate, indicating that the role of the “acylation modification network” in MIRI has expanded from the protein level to the RNA level (Figure 4).

### 3.4 Arrhythmia

The mechanistic study of arrhythmias has expanded from traditional ion channel abnormalities and electrophysiological remodeling to include metabolic-sensitive post-translational modifications. Accumulating evidence indicates that acylation-related modifications such as acetylation, N-terminal acetylation, 2-hydroxyisobutyrylation (Khib), and palmitoylation constitute an upstream regulatory network in the development and progression of arrhythmias by coordinately regulating ion channel expression and membrane trafficking, gap junction stability, calcium homeostasis, mitochondrial metabolism, oxidative stress, and cell death programs (Muñoz et al., 2023). Among these, lysine acetylation/deacetylation imbalance is the best-established mechanism. In Ang II-induced mouse myocardium, upregulation of the SIRT1/PGC-1α/FNDC5 signaling pathway was demonstrated to reduce the expression of smooth muscle α-actin (α-SMA), inhibit myofibroblast activation and collagen deposition, and thereby attenuate atrial fibrosis and the occurrence of atrial fibrillation (Cong et al., 2024). In aging-related atrial fibrillation, downregulation of SIRT1 is a prominent molecular change; its deficiency increases RIPK1 acetylation, promotes MLKL phosphorylation, and activates necroptosis, further leading to atrial cell loss, inflammatory amplification, and structural remodeling, ultimately increasing susceptibility to atrial fibrillation (Wang et al., 2024). Furthermore, acetylation directly affects cardiac electrical activity: on one hand, it modulates depolarization, repolarization, and excitation-contraction coupling through regulation of Nav1.5, Ca_v channels, and related transcriptional programs; on the other hand, Cx43 expression, microtubule stability, and sarcoplasmic reticulum Ca^2+^ cycling-related proteins are also regulated by acetylation status, thereby collectively determining conduction velocity, reentry formation, and risk of triggered activity (Hund and Mohler, 2021; Greener et al., 2022). Therefore, the role of acetylation abnormalities in arrhythmias is not limited to a single ion channel but rather manifests as a multi-level imbalance involving structural, electrophysiological, and metabolic dimensions.

In addition to classical lysine acetylation, aberrant N-terminal acetylation can directly lead to heritable proarrhythmic phenotypes. NAA10 variants disrupt NatA complex stability and impair global N-terminal acetylation capacity, manifesting as prolonged action potential repolarization in patient-derived and gene-edited iPSC cardiomyocytes, accompanied by upregulation of sarcomeric proteins and downregulation of glycolysis-related proteins (Støve et al., 2021). This indicates that N-terminal acetylation imbalance not only causes QT interval prolongation but also couples repolarization defects, metabolic reprogramming, and cardiomyopathic remodeling, resulting in a more complex electromechanical instability phenotype. Similarly, Khib, as a recently recognized novel acylation modification, has also been suggested to be closely associated with metabolic reprogramming in atrial fibrillation. In atrial tissue from patients with heart valve disease, it was found that during atrial fibrillation, the level of Khib at the K418 site of HXK1, which was regulated by HDAC2, was reduced. This reduction was shown to impair the binding of HXK1 to glucose and its catalytic activity, leading to decreased production of glucose-6-phosphate and ATP. Consequently, the protein level of Kir6.2 and the current of the KATP channel were increased, and the action potential duration was shortened, thereby participating in the development of atrial fibrillation. These findings suggested that Khib might be involved in key pathological processes of atrial fibrillation by interfering with energy substrate utilization and ionic homeostasis (Støve et al., 2021). Although specific target proteins and sites require further elucidation, existing evidence indicates that Khib is not merely a metabolic concomitant phenomenon but may represent an important regulatory layer in atrial pathological remodeling.

**FIGURE 5:**
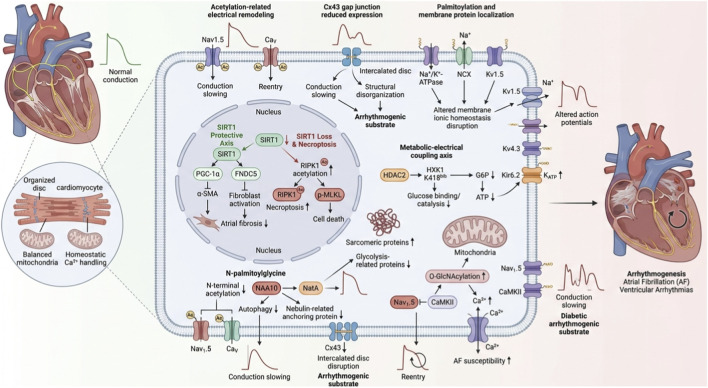
Schematic overview of how protein acylation-related modifications drive electrophysiological, structural, and metabolic remodeling in arrhythmia. The expression of α-SMA, myofibroblast activation, and collagen deposition were suppressed by the SIRT1/PGC-1α/FNDC5 signaling axis, and consequently, atrial fibrosis and the incidence of atrial fibrillation were reduced. Conversely, atrial cell loss, inflammatory amplification, and structural remodeling were induced by the downregulation of SIRT1. Nav1.5, CaV channels, and Cx43 were directly regulated by abnormal acetylation, thereby affecting heart rate. In addition to classical lysine acetylation, prolongation of action potential repolarization, accompanied by abnormal upregulation of sarcomeric proteins and downregulation of glycolysis-related proteins, was caused by defects in N-terminal acetylation mediated by NAA10/NatA. Glucose utilization was impaired and G6P and ATP production were reduced by the decreased Khib at the HXK1 K418 site regulated by HDAC2, and subsequently, Kir6.2 and KATP channel currents were upregulated, leading to atrial fibrillation. Na+/K + -ATPase was affected by abnormal palmitoylation, and consequently, Na+ and Ca2+ homeostasis as well as the repolarization process were altered. Atrial fibrillation susceptibility was also increased by N-palmitoylglycine through α1,2-mannosidase and autophagy inhibition. Furthermore, diabetes-related arrhythmias were induced by elevated O-GlcNAcylation.

Compared with the modifications described above, palmitoylation is particularly distinguished by its regulatory role in membrane protein localization and signaling microdomain assembly, thereby occupying a unique position in arrhythmias. Current studies suggest that the palmitoylation status of Na^+^/K^+^-ATPase, the Na^+^/Ca^2+^ exchanger (NCX), and Kv channel-associated proteins can affect their membrane localization, inactivation behavior, and surface expression, thereby altering transmembrane Na^+^ and Ca^2+^ homeostasis and further influencing action potential duration and the formation of triggered activity (Zhao et al., 2023; Gök et al., 2021). In addition, in rats subjected to chronic unpredictable mild stress (CUMS)-induced depression and in human samples, N-palmitoyl glycine was shown to upregulate the expression of α1,2-mannosidase, which in turn inhibited autophagy and reduced the synthesis of nebulin-related anchoring protein in cardiomyocytes, leading to intercalated disc rupture and consequently increasing susceptibility to atrial fibrillation (Chen J. et al., 2025). Notably, palmitoylation of the cytoplasmic loop of NCX was demonstrated to regulate its endocytosis and functional inhibition under stress conditions, whereas S-acylation of Kv1.5 and Kv4.3-associated proteins was found to directly participate in atrial and ventricular repolarization. Therefore, palmitoylation, through its effects on membrane protein localization and ion transport remodeling, was recognized as an important modification linking microstructural abnormalities of the cell membrane to arrhythmic phenotypes (Congreve et al., 2024). O-GlcNAcylation was also found to play a significant pathogenic role in diabetes-associated arrhythmias. Elevated myocardial O-GlcNAc levels were shown to be associated not only with collagen deposition, autophagic abnormalities, and mitochondrial dysfunction but also with altered conduction and excitability through effects on key electrophysiological proteins such as Nav1.5 (Erickson et al., 2021). In light of recent studies, this process was suggested to be further amplified by crosstalk with CaMKII activation, oxidative stress, and abnormal intracellular Ca^2+^ handling, ultimately creating a pro-arrhythmic microenvironment (characterized by slowed conduction, ectopic activity, and reentry) in diabetes (Pei et al., 2024) (Figure 5).

## 4 Therapeutic strategies targeting protein acylation in cardiovascular diseases

### 4.1 Protein acetylation targets in cardiovascular diseases

Protein acylation-related molecules are gradually transitioning from being “pathological mechanism participants” in cardiovascular diseases to therapeutic targets with translational potential. Overall, acylation targets in cardiovascular diseases are not limited to a single modified protein but encompass a complete biological process involving modifying enzymes, substrate proteins, and metabolites. One category comprises upstream acylation-regulating enzymes, such as the SIRT family, HDACs, and acetyltransferases, which determine the global acylation status of histones and non-histone proteins. Another category includes functional substrates directly regulated by acylation, such as RIPK1, Snail1, NLRP3, ion channels, and metabolic enzymes, which participate in core pathological processes including inflammatory activation, necroptosis, metabolic reprogramming, and electrophysiological remodeling. A third category involves metabolic nodes related to acyl donors such as acetyl-CoA, which influence cellular epigenetic status and stress responses by altering acyl donor availability (Drazic et al., 2021; Narita et al., 2019). Therefore, therapeutic strategies targeting protein acylation should extend from single enzyme inhibition or activation toward precise regulation in the context of specific cell types, specific substrate sites, and specific metabolic backgrounds (Table 2).

**TABLE 2:** Protein acylation-related targets in cardiovascular diseases.

Target/Molecule	Acylation type	Diseases	Mechanism	References
SIRT1	Deacetylation regulation	Heart failure, atrial fibrillation, ischemic myocardial injury	As an NAD^+^-dependent deacetylase, SIRT1 regulates the acetylation status of histone and non-histone proteins; suppresses NF-κB p65 signaling; and modulates RIPK1 acetylation and the necroptotic pathway	Kane and Sinclair (2020), Ali et al. (2018)
HDAC family (Class I/II)	Deacetylation regulation	Dilated cardiomyopathy, myocardial injury, cardiac remodeling	Regulates histone and non-histone acetylation, thereby influencing chromatin accessibility, pathological transcriptional reprogramming, and repair-related cellular phenotypes	Ali et al. (2018), Kouzarides (2007)
Acetyl-CoA metabolic axis	Regulation of acetyl-donor availability	Post-myocardial infarction repair, ischemic heart disease	Determines acetyl-group availability and thereby affects histone acetylation levels and repair-associated gene expression	Shi and Tu (2020), Lei et al. (2021)
RIPK1	Acetylation	Aging-related atrial fibrillation	Downregulation of SIRT1 increases RIPK1 acetylation, which promotes MLKL phosphorylation and activates necroptosis	Ali et al. (2018)
Snail1	Lactylation/acetylation	Post-myocardial infarction inflammation and repair	Altered acylation status affects its transcriptional activity, thereby regulating macrophage polarization and the transition from inflammation to repair	Chen S. et al. (2025)
NLRP3	Palmitoylation	Septic cardiomyopathy, inflammatory myocardial injury	Palmitoylation status influences activation/inactivation of the NLRP3 inflammasome and downstream inflammatory signaling	Zhu et al. (2024)
HXK1	2-Hydroxyisobutyrylation (Khib)	Atrial fibrillation	May affect glucose metabolic flux and energy supply as a key initiating enzyme in glycolysis	Xu et al. (2024)
TPIS	2-Hydroxyisobutyrylation (Khib)	Atrial fibrillation	Participates in the conversion of glycolytic intermediates and may affect enzymatic activity and metabolic homeostasis	Xu et al. (2024)
PGM1	2-Hydroxyisobutyrylation (Khib)	Atrial fibrillation	Participates in glucose metabolism and may influence metabolic reprogramming	Xu et al. (2024)
MLKL pathway-related molecules	Indirectly regulated by acetylation	Atrial fibrillation	Increased RIPK1 acetylation promotes MLKL phosphorylation and triggers necroptosis	Lin B. et al. (2020)

Among these targets, SIRT1 is a well-established cardioprotective deacetylase with substantial evidence. As an NAD^+^-dependent deacetylase, SIRT1 participates in inflammation suppression, oxidative stress defense, cell death regulation, and metabolic homeostasis maintenance by modulating substrates such as NF-κB, p53, FOXO, and PGC-1α (Kane and Sinclair, 2020; Sosnowska et al., 2022). SIRT1 ameliorates heart failure through the NF-κB p65/miR-155/BDNF signaling axis, suggesting that it not only functions as a deacetylase but also serves as a key upstream node in the inflammatory transcriptional network and myocardial remodeling process (Lin Y. et al., 2020). Furthermore, in aging-related atrial fibrillation, downregulation of SIRT1 leads to increased acetylation of RIPK1, which subsequently activates MLKL-dependent necroptosis, driving atrial enlargement and enhancing susceptibility to atrial fibrillation (Yang et al., 2024; Dinislam et al., 2026). Therefore, the therapeutic value of SIRT1 lies in its ability to simultaneously intervene in multiple pathological dimensions, including inflammation, cell death, metabolic abnormalities, and electrical remodeling.

The HDAC family also represents one of the most representative drug targets in cardiovascular acylation therapy. Classical HDACs regulate chromatin accessibility, stress gene expression, and cellular phenotypic switching by removing acetyl groups from lysine residues on histones and non-histone proteins (McKinsey, 2021). In cardiac hypertrophy, fibrosis, inflammatory responses, and contractile dysfunction, HDAC-mediated transcriptional remodeling is considered a key driver of pathological cardiac remodeling (Glezeva and Baugh, 2022). Studies on mesenchymal stromal cells derived from dilated cardiac tissues have also shown that class I and class II HDAC inhibitors can modulate the biological properties of repair-related cells, suggesting that HDAC-targeted intervention may not only affect cardiomyocytes themselves but also reshape the reparative microenvironment following cardiac injury (Mikšiūnas et al., 2021). Nevertheless, given that different HDAC isoforms exhibit cell type- and disease stage-specificity in the cardiovascular system, future efforts should focus on developing isoform-selective and temporally specific intervention strategies.

In addition to modifying enzymes, acyl donor metabolism also represents a noteworthy therapeutic entry point. Acetyl-CoA is the core substrate for acetylation reactions, and its generation, transport, and distribution directly influence histone acetylation levels and post-injury gene expression programs (Shi and Tu, 2020). Several studies have shown that enhancing acetyl-CoA supply can increase histone acetylation levels, activate repair- and antioxidant-related transcriptional programs following cardiac injury, and thereby improve cardiac repair (Lei et al., 2021). This finding suggests that acetylation is not solely determined by modifying enzymes; metabolic substrate availability itself can shape the epigenetic state of cardiac cells. Substrates associated with novel acylation modifications also provide new directions for disease-specific intervention. Khib exhibits significant alterations in atrial tissues from patients with atrial fibrillation, with differentially modified sites predominantly enriched in glycolysis- and energy metabolism-related proteins such as HXK1, TPIS, and PGM1 (Xu et al., 2024). Although these proteins are not traditional electrophysiological molecules, their modification status may indirectly promote atrial electrical and structural remodeling by altering energy supply, mitochondrial metabolism, and oxidative stress levels. Therefore, Khib-related metabolic enzymes and specific sites may represent novel acylation targets in the metabolic reprogramming of atrial fibrillation. Furthermore, acylation modifications can also influence cardiovascular injury repair by regulating immune cell phenotypes and inflammasome activity. On the other hand, the NLRP3 inflammasome is a key effector target in inflammatory cardiovascular diseases such as septic cardiomyopathy, myocardial ischemia-reperfusion injury, and atherosclerosis. Promoting palmitoylation of NLRP3 can inhibit its inflammasome activation and alleviate oxidative stress and inflammatory injury in septic cardiomyopathy (Zhu et al., 2024).

### 4.2 Targeted treatment for protein acetylation

As the role of protein acetylation in the occurrence and development of cardiovascular diseases has been increasingly revealed, drug intervention based on acetylation modification has gradually become an important direction in precise cardiovascular treatment. Unlike traditional drugs that mainly target hemodynamic abnormalities, neurohumoral activation, or a single ion channel, the therapeutic strategy targeting protein acetylation places greater emphasis on implementing interventions at upstream levels such as epigenetic regulation, metabolic reprogramming, inflammation amplification, and cell function determination. These therapeutic strategies can be broadly classified into drugs that regulate the levels of histone and non-histone acetylation; natural compounds that act on deacetylases or acetylation-related signaling pathways; metabolic intervention methods that indirectly reshape the acetylation state by influencing the supply of metabolites; and candidate drugs targeting novel acetylation modifications such as lactoylation and palmitoylation-related targets (Table 3).

**TABLE 3:** Cardiovascular disease drug treatments targeting protein acetylation.

Drug	Acylation type	Diseases	Mechanism	References
Resveratrol	Deacetylation	Heart failure, aging-related atrial fibrillation	Activates SIRT1, suppresses inflammatory signaling and RIPK1 acetylation-related necroptosis	Ali et al. (2018)
Class I/II HDAC inhibitors	Acetylation/deacetylation	Dilated cardiomyopathy, myocardial injury	Reverse pathological transcriptional programs and regulate repair-related cellular functions and gene expression	Ali et al. (2018), Kouzarides (2007)
Acetyl-CoA-promoting metabolic interventions	Acetylation	Myocardial infarction	Increase acetyl-CoA supply, enhance histone acetylation, and activate repair-related gene expression	Shi and Tu (2020), Lei et al. (2021)
Astragaloside IV	Lactylation/acetylation	Myocardial infarction	Regulates Snail1 lactylation and acetylation to mediate macrophage polarization	Chen S. et al. (2025)
Vaccarin	Palmitoylation	Septic cardiomyopathy	Enhances NLRP3 palmitoylation and promotes its inactivation, thereby suppressing inflammasome activation	Zhu et al. (2024)
Eugenol	Acetylation	Ischemic myocardial injury	Maintains H3K27ac levels under ischemic conditions	Randhawa et al. (2023)
Apigenin	Acetylation	Ischemic myocardial injury	Maintains the active histone mark H3K27ac	Randhawa et al. (2023)
Bis-demethoxy curcumin	Acetylation	Ischemic myocardial injury	Preserves active histone acetylation marks under ischemic conditions	Randhawa et al. (2023)
D-gamma-tocopherol	Acetylation	Ischemic myocardial injury	Helps maintain H3K27ac levels	Randhawa et al. (2023)
Ambroxol	Acetylation	Ischemic myocardial injury	Maintains active histone acetylation marks	Randhawa et al. (2023)
L-Ergothioneine	Acetylation	Ischemic myocardial injury	Preserves relative stability of H3K27ac under ischemic stress	Randhawa et al. (2023)
Ciclopirox ethanolamine	Acetylation	Ischemic myocardial injury	Maintains active histone acetylation marks	Randhawa et al. (2023)
Tanshinone IIA	Acetylation	Ischemic myocardial injury	Reduces the decline of H3K27ac under ischemic conditions	Randhawa et al. (2023)
Shenfuyixin Granules	Deacetylation	Myocardial infarction	Regulates protein deacetylation through the SIRT3/FOXO1 axis, enhances mitophagy, and alleviates post-infarction myocardial injury	Guo et al. (2025)
Lithospermic acid	Deacetylation	Myocardial injury	Alleviates cardiomyocyte injury and cardiomyopathy progression through SIRT3-mediated p53 deacetylation	Zhao et al. (2026)

Under ischemic and hypoxic conditions, the histone acetylation status of cardiomyocytes undergoes significant alterations, thereby affecting the expression of injury-responsive and repair-related genes. In cardiomyocytes exposed to ischemic conditions, the active histone marker H3K27ac is markedly decreased, whereas the repressive marker H3K27me3 is significantly increased, suggesting that myocardial ischemia induces an unfavorable epigenetic remodeling. Based on this, high-throughput drug screening has identified multiple compounds capable of maintaining H3K27ac levels, including the phenolic compounds eugenol, apigenin, resveratrol, bis-demethoxy curcumin, D-gamma-tocopherol, ambroxol, as well as the non-phenolic compounds L-ergothioneine, ciclopirox ethanolamine, and Tanshinone IIA (Randhawa et al., 2023). The common feature of these agents is their ability to preserve a favorable histone acetylation status under ischemic stress, thereby protecting cardiomyocyte transcriptional activity and stress adaptability. Although the specific molecular mechanisms of each drug have not been individually elucidated, the overall implication is clear: modulating key histone acetylation marks such as H3K27ac could serve as an important strategy against ischemic myocardial injury. In addition to directly targeting acylating enzymes, regulating acyl donor metabolism represents another important pharmacological approach for targeting protein acylation. A study by Lei et al. demonstrated that specific metabolites can increase acetyl-CoA generation and improve cardiac repair after myocardial infarction by promoting histone acetylation (Chen X. et al., 2025). This study suggests that post-infarction cardiac regeneration and repair depend not only on inflammation control and fibrosis extent but also on whether cells can establish a favorable epigenetic program, a process closely linked to acetyl-CoA availability.

SIRT1 is one of the most representative protective targets in cardiovascular acylation regulation, and therefore drug strategies aimed at activating SIRT1 have garnered substantial research interest. SIRT1 can ameliorate heart failure by modulating the NF-κB p65/microRNA-155/BDNF signaling cascade (Lin B. et al., 2020). This suggests that enhancing SIRT1 activity can alleviate chronic cardiac inflammation, improve neuroregulatory abnormalities, and limit the progression of myocardial remodeling. The natural polyphenolic compound resveratrol, a well-known natural product, can reduce aging-related atrial fibrillation susceptibility by activating atrial SIRT1 and inhibiting RIPK1 acetylation-mediated necroptosis (Fang et al., 2022). HDAC inhibitors represent the most well-established class of agents in protein acylation-based therapy. Class I and class II HDAC inhibitors can act as therapeutic modulators of mesenchymal stromal cells derived from dilated cardiac tissue, suggesting that they not only act on cardiomyocytes but may also improve the cardiac disease microenvironment by regulating the phenotype and secretory function of repair-related cells (Yan et al., 2024; Xu et al., 2020; Kashyap et al., 2021). HDAC inhibitors also exhibit effects in attenuating injury, improving cell survival, and promoting protective transcriptional activation in myocardial ischemia-reperfusion injury. However, these drugs may cause side effects such as potassium channel remodeling and action potential prolongation. Nevertheless, HDAC inhibitors remain central to cardiovascular disease treatment research as a representative drug class in protein acylation therapy. With the deepening of research into novel acylation modifications, interventions targeting lactylation have begun to enter the scope of cardiovascular disease investigation. A study by Chen et al. showed that astragaloside IV can regulate the lactylation and acetylation status of Snail1, thereby mediating macrophage polarization and improving myocardial infarction (Chen S. et al., 2025). This reflects that the targets of protein acylation-based therapy have expanded from traditional cardiomyocytes to key regulatory cells in the post-injury immune microenvironment. The polarization state of macrophages directly determines the efficiency of inflammatory clearance and the quality of subsequent repair after myocardial infarction. In addition to acetylation and lactylation, palmitoylation-related drug strategies are also beginning to show potential. Research by Zhu et al. demonstrated that vaccarin can attenuate cardiac oxidative stress and inflammatory response in septic cardiomyopathy by enhancing NLRP3 palmitoylation and thereby inactivating it (Hou et al., 2024). Furthermore, the traditional Chinese medicine compound Shenfuyixin Granules and natural product Lithospermic acid can also regulate the deacetylation of CPT2 and SIRT3, thereby inhibiting the excessive oxidation of fatty acids in the heart and achieving the effect of preventing and treating diabetic cardiomyopathy (Guo et al., 2025; Zhao et al., 2026). In septic cardiomyopathy, excessive activation of the NLRP3 inflammasome is an important mechanism leading to myocardial depression, cytokine storm, and cellular injury. Vaccarin achieves inactivation by promoting NLRP3 palmitoylation, indicating that palmitoylation is not always an activating modification; its specific effect is determined by the substrate and the modification environment.

## 5 Summary

Protein acylation serves as an important molecular link connecting metabolic reprogramming, epigenetic regulation, and alterations in protein function, and has emerged as a key direction for deciphering cardiovascular disease mechanisms and developing novel therapeutic strategies. Modifications such as acetylation, lactylation, 2-hydroxyisobutyrylation, and palmitoylation are widely involved in processes including myocardial ischemia-reperfusion injury, myocardial infarction, heart failure, atrial fibrillation, and septic cardiomyopathy, with their effects spanning multiple pathological aspects such as inflammatory responses, cell death, mitochondrial metabolism, immune cell polarization, ion channel homeostasis, and myocardial remodeling. Currently, SIRT1, the HDAC family, the acetyl-CoA metabolic axis, Snail1, NLRP3, and Khib-related metabolic enzymes represent promising acylation-regulating targets. Intervention strategies targeting these nodes—such as maintaining histone acetylation homeostasis, activating SIRT1, inhibiting HDACs, enhancing acetyl-CoA supply, and modulating Snail1 lactylation/acetylation and NLRP3 palmitoylation—have shown potential in ameliorating myocardial injury, inflammatory responses, cardiac remodeling, and repair outcomes.

Nevertheless, despite rapid progress in this field, several key issues remain to be addressed. First, different acylation modifications are not independent of each other but exhibit extensive cross-regulation. How to systematically dissect the synergistic, antagonistic, and hierarchical relationships among various acylations remains an important direction for future research. Second, acylation modifications possess pronounced spatiotemporal and cell-type specificity. The same modification may produce entirely different functional consequences in cardiomyocytes, fibroblasts, endothelial cells, and immune cells; similarly, the role of a given target may differ between the acute injury phase and the chronic remodeling phase. Therefore, future studies will need to increasingly rely on technologies such as single-cell omics, spatial transcriptomics, quantitative modification omics, and high-resolution proteomics to achieve precise mapping of acylation events. Moreover, most current studies remain at the level of basic experiments and animal models, with relatively insufficient clinical translational evidence. On one hand, broad-spectrum acylation-modulating drugs often carry off-target effects and systemic side effects, potentially causing electrophysiological abnormalities or other organ toxicities. On the other hand, although natural active compounds exhibit favorable multi-target regulatory properties, they face challenges such as low bioavailability, complex mechanisms of action, and insufficient pharmacokinetic stability. Future drug development should place greater emphasis on target selectivity, tissue specificity, and optimization of administration routes, with particular need for precision intervention strategies targeting specific acylating enzymes, specific modification sites, or specific cell populations. At the same time, establishing a more comprehensive acylation biomarker panel for cardiovascular diseases will facilitate patient stratification, therapeutic monitoring, and prognostic assessment.

Overall, research on protein acylation is driving a shift in cardiovascular disease treatment from simply blocking downstream injury toward remodeling upstream regulatory networks. As key targets and druggable mechanisms continue to be clarified, targeting protein acylation holds promise as an important direction for precision cardiovascular therapy.
